# Mobile health for mental health support: a survey of attitudes and concerns among mental health professionals in Poland over the period 2020-2023

**DOI:** 10.3389/fpsyt.2024.1303878

**Published:** 2024-03-15

**Authors:** Monika Dominiak, Adam Gędek, Anna Z. Antosik, Paweł Mierzejewski

**Affiliations:** ^1^ Department of Pharmacology, Institute of Psychiatry and Neurology, Warsaw, Poland; ^2^ Praski Hospital, Warsaw, Poland; ^3^ Department of Psychiatry, Faculty of Medicine, Collegium Medicum, Cardinal Wyszynski University, Warsaw, Poland

**Keywords:** mHealth, mobile health, telehealth, digital health, smartphone, APP, acceptance, expectation

## Abstract

**Introduction:**

Mobile health (mHealth) has emerged as a dynamic sector supported by technological advances and the COVID-19 pandemic and have become increasingly applied in the field of mental health.

**Aim:**

The aim of this study was to assess the attitudes, expectations, and concerns of mental health professionals, including psychiatrists, psychologists, and psychotherapists, towards mHealth, in particular mobile health self-management tools and telepsychiatry in Poland.

**Material and methods:**

This was a survey conducted between 2020 and 2023. A questionnaire was administered to 148 mental health professionals, covering aspects such as telepsychiatry, mobile mental health tools, and digital devices.

**Results:**

The majority of professionals expressed readiness to use telepsychiatry, with a peak in interest during the COVID-19 pandemic, followed by a gradual decline from 2022. Concerns about telepsychiatry were reported by a quarter of respondents, mainly related to difficulties in correctly assessing the patient’s condition, and technical issues. Mobile health tools were positively viewed by professionals, with 86% believing they could support patients in managing mental health and 74% declaring they would recommend patients to use them. Nevertheless, 29% expressed concerns about the effectiveness and data security of such tools. Notably, the study highlighted a growing readiness among mental health professionals to use new digital technologies, reaching 84% in 2023.

**Conclusion:**

These findings emphasize the importance of addressing concerns and designing evidence-based mHealth solutions to ensure long-term acceptance and effectiveness in mental healthcare. Additionally, the study highlights the need for ongoing regulatory efforts to safeguard patient data and privacy in the evolving digital health landscape.

## 1 Introduction

Over the last few years, mobile health (mHealth) has been one of the most dynamic sectors of medicine. Initially, mass internet access and then smartphones, which offer mobile applications, enabled this trend. Research to date suggest that mobile apps applied in mental health can support the diagnosis of mental disorders, psychoeducation, provide various forms of psychotherapy, or facilitate contact with a specialist, including serious mental illnesses such as bipolar disorder or schizophrenia (1, 2) and health crisis such as suicide risk (3). Smartphones also allow patients with mental disorders to be monitored continuously, in real time (4, 5). These solutions can both provide a complementary form of patient care, but also enable a personalized approach to the patient.

An important stimulus for the development of mHealth in mental health was the COVID-19 pandemic, which made the traditional form of contact with a doctor impossible. In particular, there has been a sharp increase in the use of two solutions offered by mHealth, namely telepsychiatry and mHealth self-management tools (especially mobile apps) in psychiatric care compared to previous years (6, 7). At the same time, this contributed to an increase in mental distress and psychiatric symptoms in the community during this period (8–10). The pandemic has therefore forced a transformation of healthcare and the spread of these solutions, but they can still be widely use afterwards. However, the use of telemedicine (telepsychiatry in the field of mental health) to treat patients is nothing new. It was first used in psychiatry in 1959 and mainly served geographically isolated populations. This is what is currently known as telepsychiatry, which is defined as ‘the delivery of psychiatric assessments or follow-up interviews from a distance using technologies such as telephone calls, audio and video digital platforms, and healthcare monitoring devices’ (11).

Mobile Health is a rapidly growing field that use the capabilities of mobile devices such as smartphones, patient monitoring devices, personal digital assistants (PDAs), and other wireless devices to enhance healthcare services (12). It extends the reach of medical care by enabling remote monitoring and consultations, making healthcare more accessible. Furthermore, mHealth allows individuals to manage their health actively through apps and wearable technology (12). As this solution evolves, it has the potential to revolutionize healthcare delivery and improve patient outcomes while also presenting challenges related to privacy and regulation that need careful consideration (13). In the mental health realm, mHealth offers innovative solutions such as internet-based therapies, text messaging for psychiatric services, and smartphone apps for monitoring and treating various psychiatric conditions including depression, bipolar disorder, schizophrenia and other health crises (1, 3, 14–17). These technologies improve clinical outcomes, but also reduce stigma and improve access to care, while initiatives like SMS campaigns, community outreach, and medication tracking further strengthen mental health support (18). As mobile technology advances, mHealth plays a crucial role in expanding access to quality mental healthcare.

Several factors will determine whether mobile solutions in mental health will become widely implemented. Firstly, there is a need for reliable, validated apps and websites that can truly help patients (19–21). The perception of such solutions by people with mental illnesses is also extremely important, as this may determine their ultimate use (22, 23). Studies conducted on this group showed high satisfaction with tested interventions (23, 24). Notably, studies emphasized that user interest in these solutions decreased over time. A Polish study using a mobile app for monitoring BD patients observed a 44% dropout rate at one year (25). In studies conducted to date, patients emphasized the necessity for approaches that are tailored to their preferences and needs, characterized by user-friendliness and genuine helpfulness. Negative aspects included continuous reminders, a sense of being monitored, and loss of dignity and autonomy (22, 26, 27). A key, but often ignored and consequently much under-researched perspective is how mental health professionals view such solutions (28). Although some solutions are already implemented, especially in the therapeutic process (29), and research to date indicates that mental health professionals are aware of their existence, they rarely use them (30, 31). One pre-pandemic study (2019) found that professionals (psychiatrists and psychotherapists) know significantly less about mental health apps than patients (30). Only 33.7% of experts were familiar with at least one e-mental health app and 8.7% had tried it. However, more clinicians were advocates than sceptics of these solutions 68.3% vs 29.8% (30). They also believe that these solutions will become more important in the future (30). Time has shown that we did not have to wait long. After the outbreak of the pandemic, the market for mental health apps grew exponentially, and the subject of consideration was not what professionals thought of them, but whether they should prescribe them to patients (31). In a 2022 Portuguese survey of 160 clinician, mainly psychologists but also psychiatrists, as many as 87.2% supported the possibility of prescribing mental health apps (31).

Regarding professionals’ perceptions of telepsychiatry, three surveys at the beginning of the pandemic (2020) conducted in the US (32, 33) and the UK (34) indicated high levels of satisfaction with video consultations. Furthermore, 95.5% of clinicians responded that they would like telepychiatric visits to make up at least 25% of their practice in the future (33). As the pandemic continued, perceptions might have changed. This is shown in a 2021-2022 multicenter study conducted in the UK and Italy (35). In general, telepsychiatry was perceived as most convenient for purpose-specific follow-up visits, such as medication checks, however, it was perceived as less effective for setting up a therapeutic relationship or assessment of mental status in acute mental crisis.

It is also important that clinicians recognize the real needs and concerns that mHealth may confront in mental health (34, 36, 37). Engaging them in the development process is therefore crucial to their use and proper application. To date, only a few studies have assessed the attitudes and concerns of mental health professionals toward mobile solutions (30, 34, 36–38), while far more attention in the literature has been given to health professionals working in other fields of medicine (39–41). No study on mental health professionals has been conducted in Poland to date either. This may seem surprising given that the mobile app market, as well as video consultations, is currently primarily concerned with mental health.

The aim of this study was to assess the attitudes and expectations towards mHealth in particular telepsychiatry (video/teleconsultations) and mobile health tools such as applications, wristbands, smart watches etc., among mental health professionals (psychiatrists, psychologists, psychotherapists) in Poland.

## 2 Materials and methods

### 2.1 Research questionnaire

A questionnaire was created taking into account the available literature, knowledge of the specificity of mental disorders and the experience of clinicians from the Institute of Psychiatry and Neurology, who have conducted Polish research implementing mHealth in the field of mental health. The research questions were related to the final shape of mHealth solutions, mainly telepsychiatry and mHealth self-management tools, that will be acceptable to professionals in the perspective of their long-term use. We sought to assess the needs, expectations and areas of application of mobile technologies from a professional perspective. Following the development of the survey questionnaire, a pilot study was conducted to assess the ease of using the questionnaire, answering the questions asked, and collecting comments on the survey instrument. For this questions on respondents' evaluation of the survey in terms of the number of questions, as well as the ease of understanding the questions and answering them. The original version of the questionnaire was tested on 18 clinicians (7 psychiatrists and 11 psychologists). According to 72.2% (n=13) of respondents, the number of questions in the questionnaire was appropriate, according to 11.1% (n=2) too few, and according to 16.7% (n=3) too many. According to 88.9% (n=16) of respondents, the questions were formulated in a clear and understandable way, while 11.1% (n=2) of respondents assessed them as somewhat too complicated. Respondents did not make significant comments on the content of the questions asked, 22.2% (n=4) respondents made technical comments regarding division of the survey into 3 separate parts. All raised issues were taken into account, discussed in the research team and resulted in the development of the final version of the survey.

The research questions were grouped into three issues (**Supplementary Material**). At the forefront of the questionnaires, there is brief information about the purpose of the survey. This is followed by research questions, both closed and open, covering the following three main areas:

1) the prevalence of mobile device and internet usage and clinicians’ current experiences with the use of mHealth solutions in the area of health and mental health (what percentage of respondents already have some experience);
2) clinicians’ attitudes, opinions, and preferences regarding mHealth solutions in mental health, in particular views regarding telepsychiatry and self-management tools. The questions relate to interest in the use of mobile technology in mental health, the opportunities that it can offer for patients, the level of readiness to use it, factors influencing clinicians’ attitudes, needs, expectations and areas of application of mHealth technology from the clinicians’ perspective;
3) concerns and risks associated with the adoption of mHealth solutions for mental health management.

The questionnaire ended with a metric collecting data on the demographic characteristics of the respondents. The survey consisted of 26 questions that could be answered on either a 3-point, 5-point Likert scale, had a choice of one of two yes/no options or were open-ended. Some questions were multiple choice. The survey was also designed in an online traditional version and for mobile users.

The study was notified to the Bioethics Committee at the Institute of Psychiatry and Neurology in Warsaw, Poland. A formal approval of the Bioethics Committee was not required as it was a questionnaire surveys not endangering the well-being and interests of the participants. Data were treated with confidentiality, equality and fairness, respecting the Helsinki principles (42).

### 2.2 Study sample and recruitment

**Figure 1:**
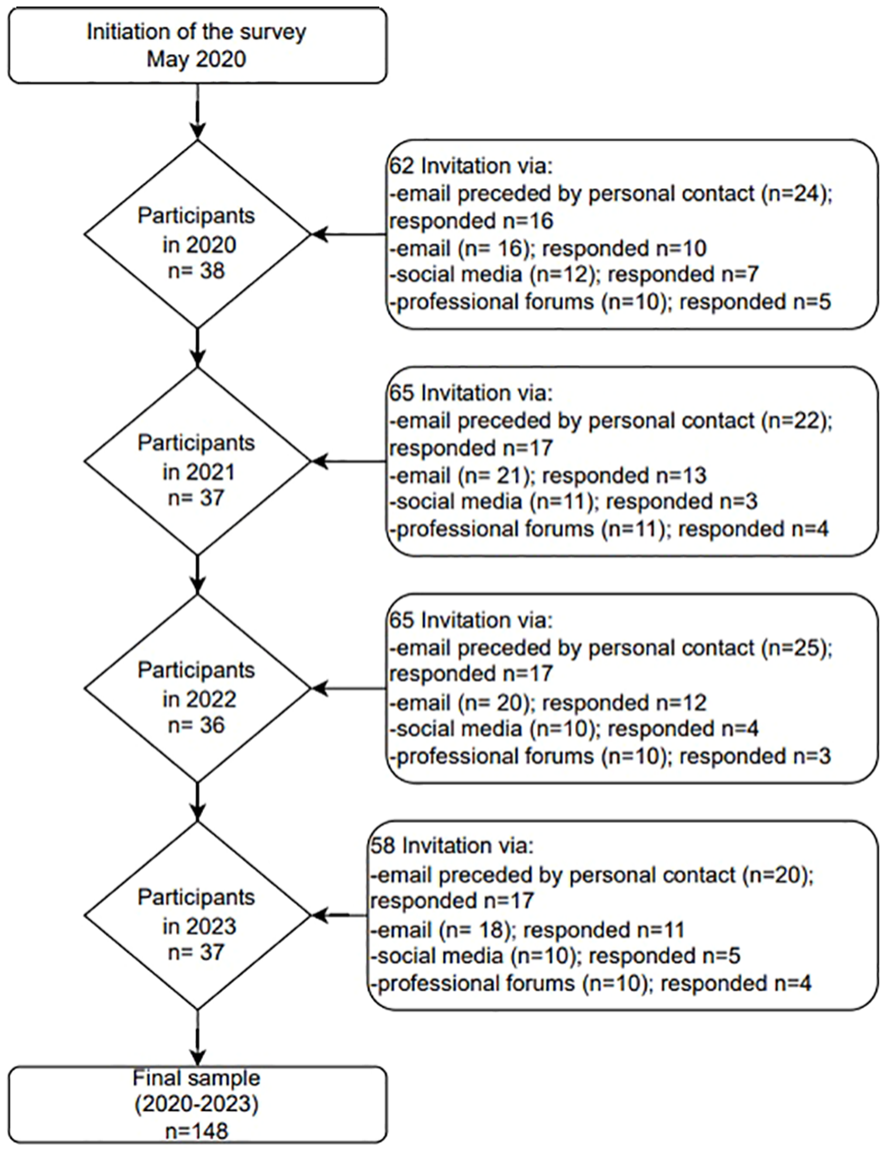
The process of including respondents in the survey.

The survey was conducted among professionals working in mental health care and involved psychiatrists, psychologists, therapists agreeing to participate in the survey. The survey was conducted on a random representative sample of Polish professionals working in mental health facilities. Individuals were recruited from a representative sample, the sample frame was a registry of all mental health facilities in 16 regions (voivodeships) in Poland. The study selected a stratified random sample with proportional allocation. The questionnaire was distributed initially to professionals working in the Institute of Psychiatry and Neurology and then sent to mental health service providers (counselling centers and hospitals) in 16 regions (voivodeships) in Poland. Information with an invitation to take part in the survey was sent out via email. To ensure diversity of the sample we recruited respondents through additional sources, including social media and forums for professionals. The distribution ran continuously from May 2020 to the end of April 2023. Over 250 invitations were sent to mental healthcare professionals. In total, 148 professionals completed the survey (response rate 59.2%). Details of how the recruitment process was conducted are included in the **Figure 1**.

### 2.3 Statistical analysis

Statistical analyses was carried out using Statistica 13.3 software. Descriptive statistics were calculated using means and standard deviations, as well as median and interquartile range - for data not meeting the criteria for normal distribution. Chi-square (χ^2^) tests were used if the variables were presented on a nominal scale and Kruskal-Wallis test if the study variables were collected on an ordinal scale and did not have a normal distribution. In the case of small group sizes, the chi-square test combined low-ranked responses, as long as this did not interfere with the interpretation of the results. For *post-hoc* comparisons, the Bonferroni correction was used to give confidence in the power of the test. Relationships between variables were verified using regression and correlation methods The influence of age, gender, specialty (doctor/psychologist/therapist), size of the town in which the specialist practices on the responses were analyzed. Differences were be assessed at an assumed statistical significance level of p<0.05.

## 3 Results

### 3.1 General characteristics of the respondents

The web-based survey was completed by 148 mental health professionals (n=148). All closed questions were completed by respondents (100%). The participants characteristics is shown in **Table 1**.

**Table 1:** General characteristics of the respondents (n=148).

Variables		Percent
Sex	Female	67%
	Male	32%
Age	25-39	27%
	40-55	56%
	55-64	15%
Education	Medical doctor	57%
	Psychologist/psychotherapist	42%
Professional activity	Outpatient clinic	59%
	Hospital/psychiatric ward + Outpatient clinic	21%
	Hospital/psychiatric ward	3%
	Private practice	14%
	General hospital + Outpatient clinic	1%
Workplace	City > 250 000	69%
	City 50 000 – 250 000	25%
	City <50 000	5%
Type of psychotherapy used by psychotherapists (n=62)	Cognitive-behavioral therapy	29%
	Integrative/holistic therapy	26%
	Interpersonal therapy	24%
	Psychodynamic therapy	13%
	Humanistic therapy	8%

### 3.2 Prevalence and usage of mHealth

#### 3.2.1 Prevalence and usage of mobile devices and mHealth solutions – aggregate analysis (2020–2023)

**Figure 2:**
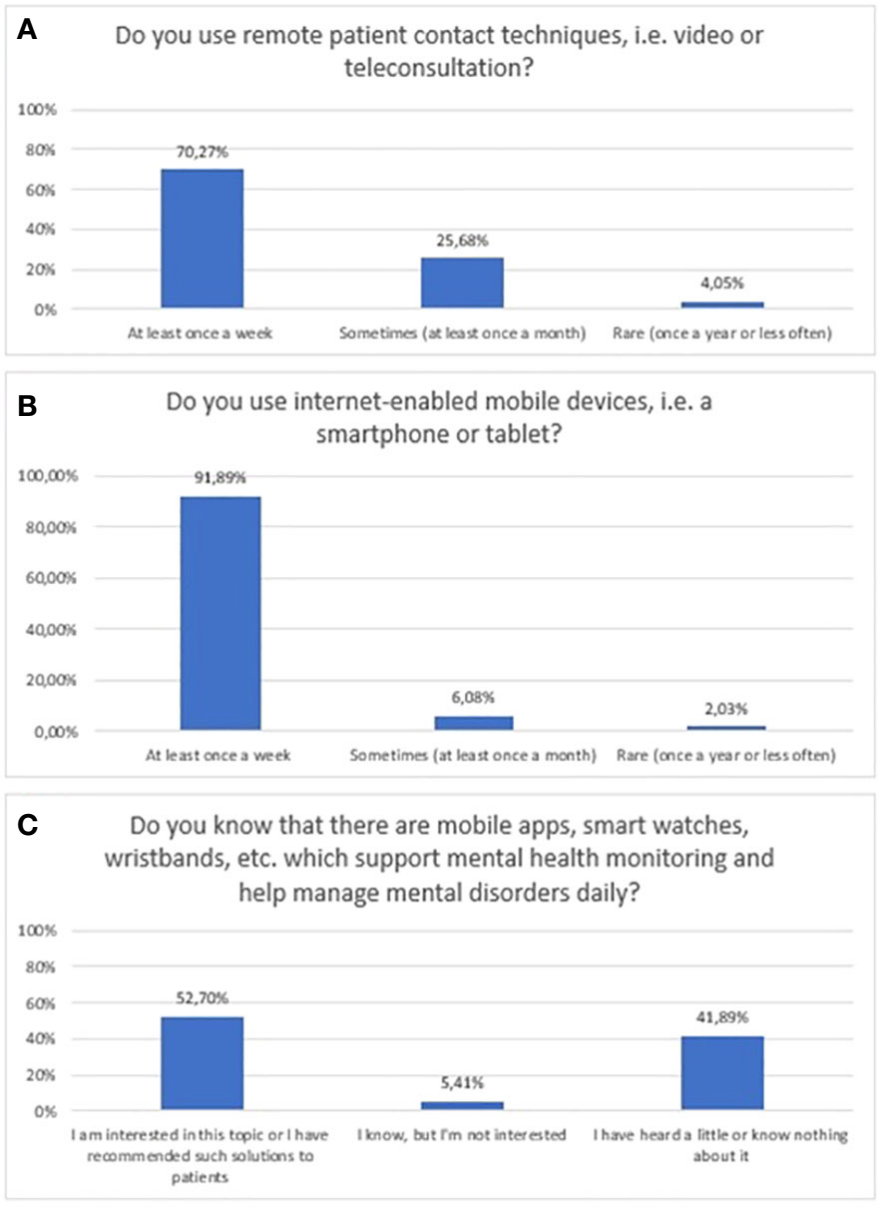
Responses regarding prevalence and usage of mHealth among mental health professionals; **(A)** Usage of remote techniques to communicate with patients; **(B)** Usage of mobile devices; **(C)** Awareness of mHealth tools in mental health.

A substantial number of respondents declared that they use mobile devices at least once a week (91.89%). In addition, the majority of them at least once a week (70.3%) use remote patient contact techniques (video/teleconsultation). Noticeably fewer respondents - 52.7% - stated that they were interested in the topic of mHealth tools, or recommended it to patients. Simultaneously, 41.9% of respondents had heard little or nothing about it. The exact distribution of responses to questions related to the prevalence and usage of new technologies is presented in **Figure 2**.

#### 3.2.2 Changes in prevalence and use of mobile devices and mHealth solutions between 2020 and 2023

**Figure 3:**
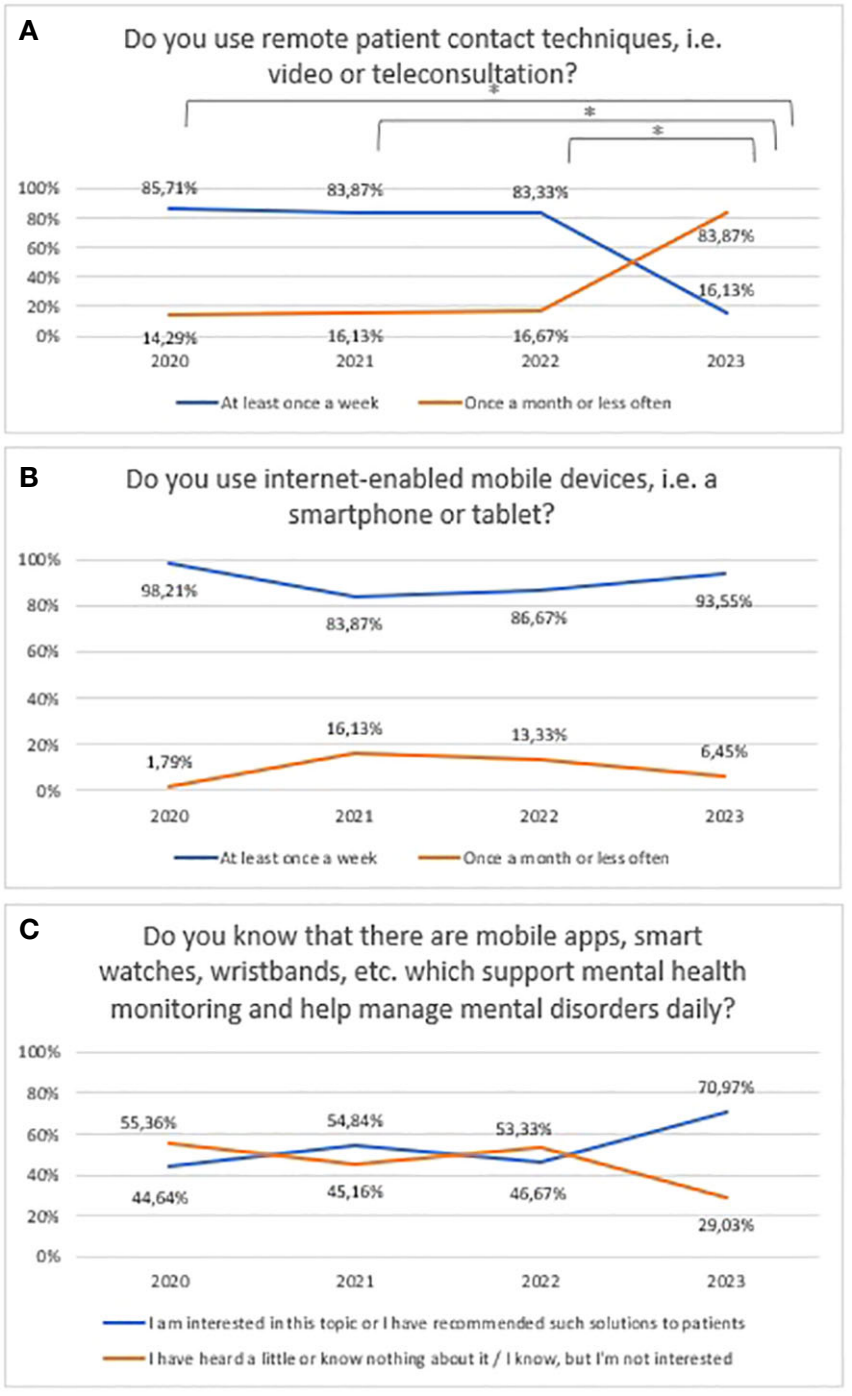
Responses regarding prevalence and usage of mHealth among mental health professionals between 2020 and 2023; **(A)** Usage of remote techniques to communicate with patients; **(B)** Usage of mobile devices; **(C)** Awareness of mHealth tools in mental health. *statistically significant difference.

Prevalence of using video/teleconsultation in mental health between 2020 and 2023 is shown in **Figure 3A** (χ^2^ = 55,08556, df=3, p<0.001; statistically significance: 2020 vs 2023: χ^2^ = 40,58, df=1, p<0.001; 2021 vs 2023: χ^2^ = 28,45, df=1, p=<0.001; 2022 vs 2023: χ^2^ = 27,55, df=1, p=<0.001). Prevalence of using mobile devices between 2020 and 2023 is shown in **Figure 3B**. The “Never”, “Rarely – once a year or less often” and “Sometimes – at least once a month” responses have been summed to ensure sufficient group numbers to perform a chi-square test. However, in **Figure 3B** χ^2^ test was impossible to implement due to the still too-small numbers in the groups. Prevalence of awareness of mHealth tools in mental health between 2020 and 2023 is shown in **Figure 3B**. The “ I know, but I’m not interested”, and “I have heard a little or know nothing about it” responses have been summed to ensure sufficient group numbers to perform a chi-square test (χ^2^ = 6,10, df=3, p=0.107).

### 3.3 Attitudes, expectations, and preferences towards telepsychiatry and mHealth tools in mental health care

#### 3.3.1 Attitudes, expectations, and preferences towards telepsychiatry and mHealth tools in mental health care - aggregate analysis (2020–2023)

**Figure 4:**
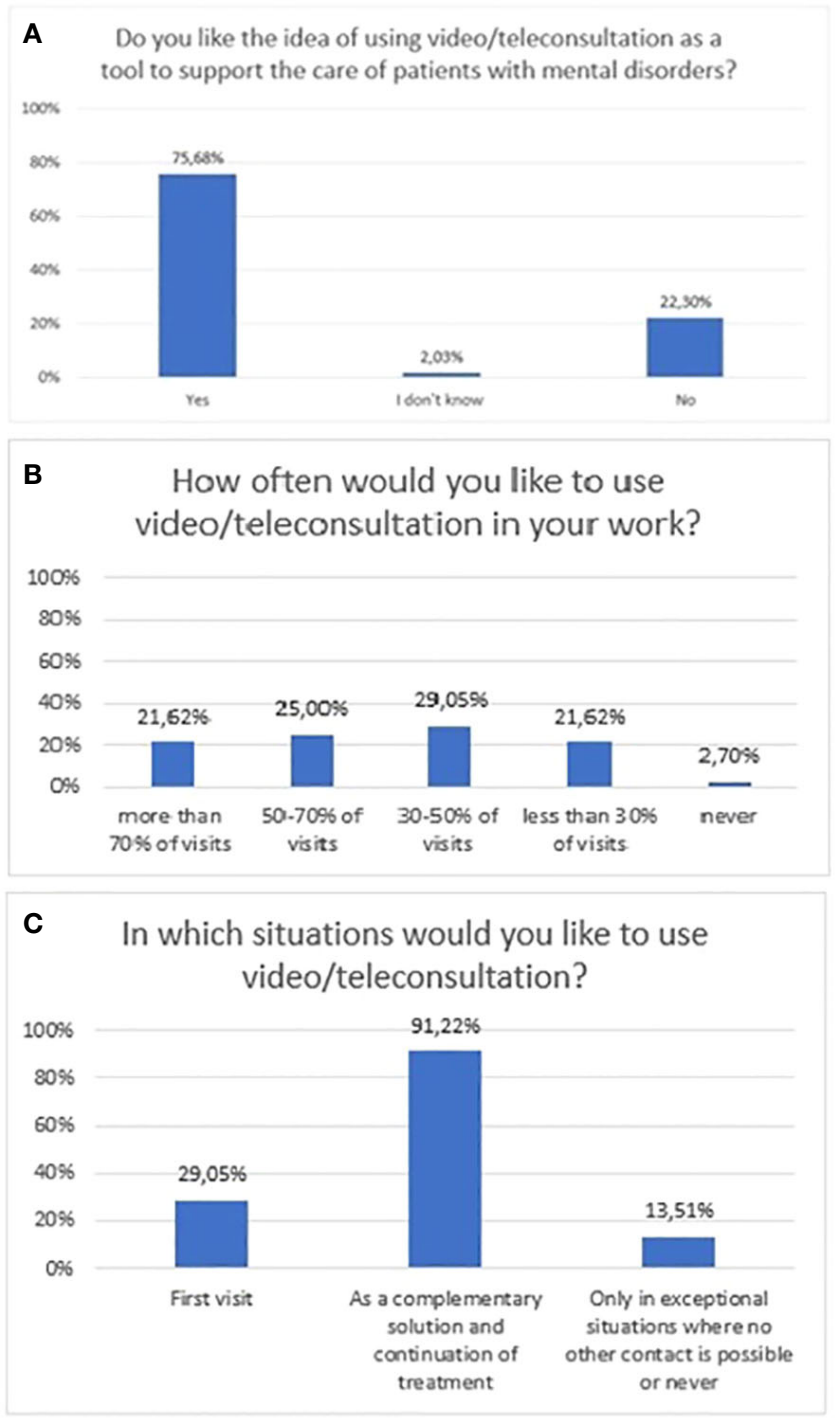
Responses regarding attitudes, expectations, and preferences section; **(A)** attitudes towards video/teleconsultation; **(B)** expected frequency of video/teleconsultation use at work; **(C)** Situations in which respondents would like to use video/teleconsultations.

The majority of respondents liked the idea of using video and teleconsultation as a support tool for patients with mental disorders (75.7%, n=112). In a multiple-choice question, the majority of respondents declared it could be applied as a complementary solution, used in the continuation of treatment (91.2%, n=135). According to a minority, 29.1% (n=43) of respondents, these can be applied at the first visit (**Figure 4**). Also, a minority of respondents would like to use remote visits more than 50% of the time - 21.6% (n=32) would like remote visits to account for 70-100% of all visits, and 25% (n=37) of respondents thought 50-70%. The exact distribution of responses to questions related to attitudes towards video/teleconsultations is shown in **Figure 4**.

**Figure 5:**
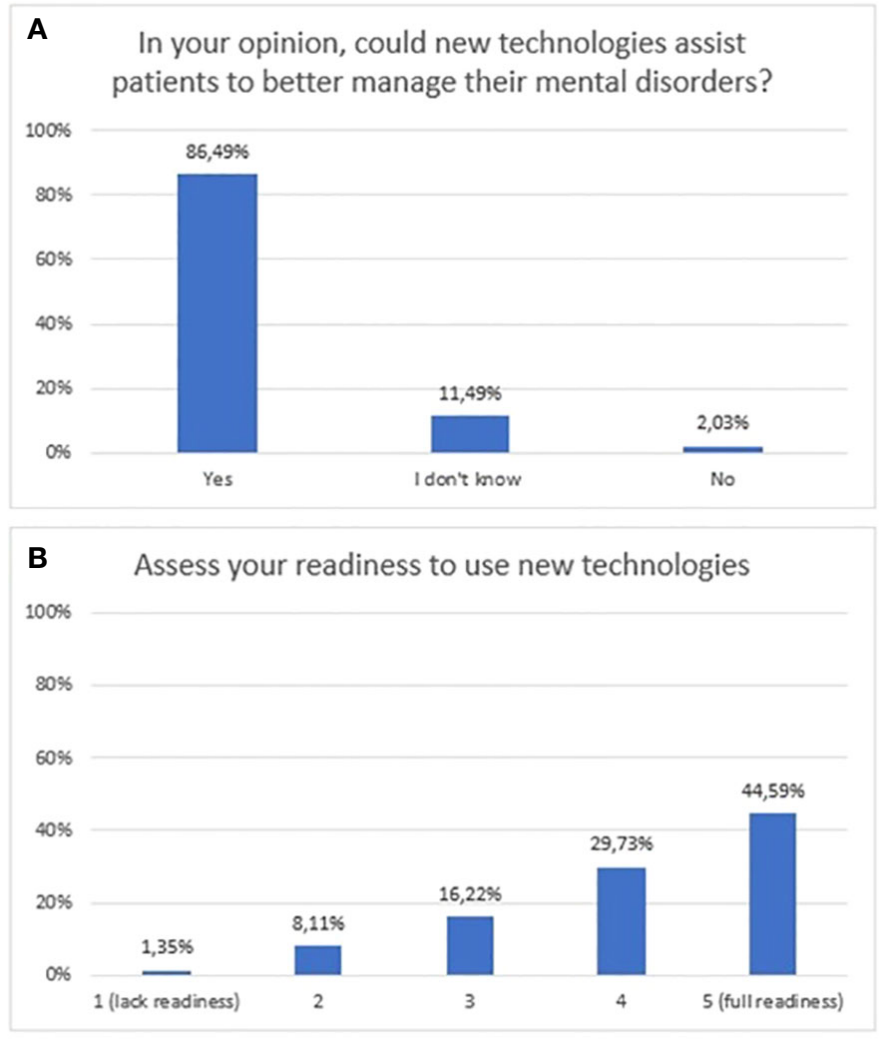
Responses regarding attitudes towards new technologies; **(A)** opinions regarding new technologies as a tool to support patients; **(B)** stated readiness to use new technologies.

According to the majority of respondents, mHealth tools can help patients to better cope with their mental illness (86.5%, n=128). Furthermore, the majority of respondents rated their readiness to use them as “4” (29.7%, n=44) or “5” (44.6%, n=66), where 5 meant full readiness. The exact distribution of responses to questions related to attitudes is shown in **Figure 5**.

#### 3.3.2 Changes in attitudes, expectations, and preferences towards telepsychiatry and mHealth tools between 2020 and 2023

**Figure 6:**
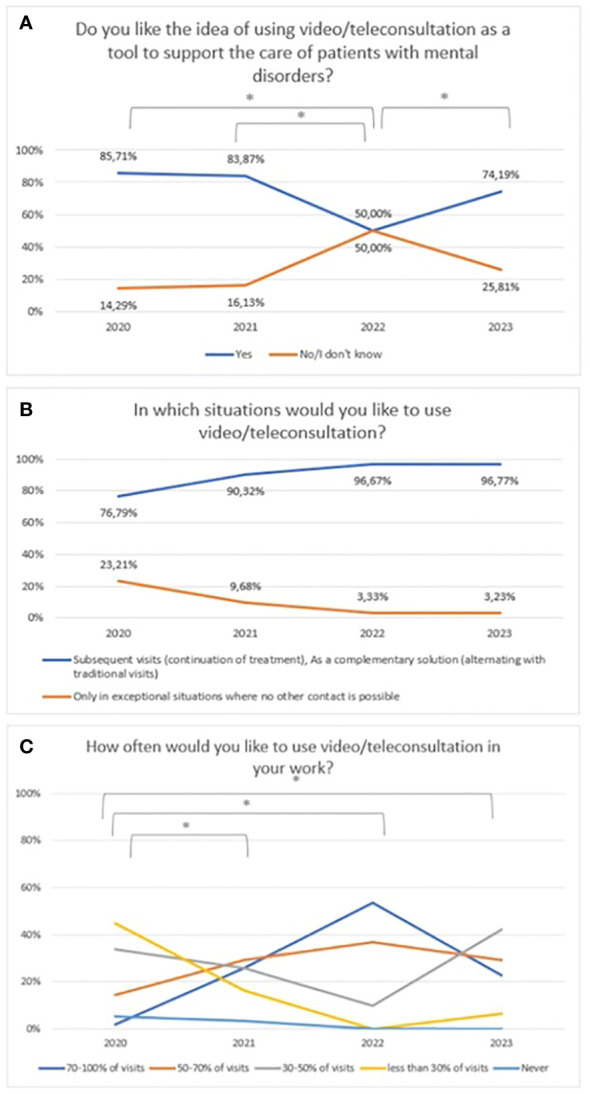
Responses regarding: **(A)** idea of using video/teleconsultation over the period 2020-2023. *statistically significant difference; **(B)** the desire to use video/teleconsultation in specific situations over the period 2020-2023; **(C)** the declared desire for frequency of use video/teleconsultation over the period 2020-2023. *statistically significant difference.

A statistically significant decrease was found between 2020 and 2022 regarding attitudes towards video/teleconsultation (χ^2^ = 14,98, df=3, p=0.002). The Bonferroni correction was included in the comparisons for each year (statistically significance: 2020 vs 2022: χ^2^ = 12,72, df=1, p=<0.001; 2021 vs 2022: χ^2^ = 7,94, df=1, p=0.005; 2022 vs 2023: χ^2^ = 14,98, df=3, p=0.002). The trend is presented in **Figure 6**. From 2022 onwards, there is an upward trend, but in 2023 not statistically significant when compared to the baseline assessment in 2020, which is when the pandemic began. Over the period 2020-2023, there was an upward trend but not a statistically significant in the preference for the use of video/teleconsultation in certain situations (firs/subsequent visits) (**Figure 6**). Changes in the declared frequency to use video/teleconsultation frequency are shown in **Figure 6**. The variable was tested on an ordinal scale and the Kruskalla-Wallis test showed statistical significance (H=27.70, p<0.001). The greatest difference between years relates to the shift in the declared intention to use view/teleconsultation starting in 2022 from over 50% of all visits towards a frequency of less than half of all visits in 2023.

**Figure 7:**
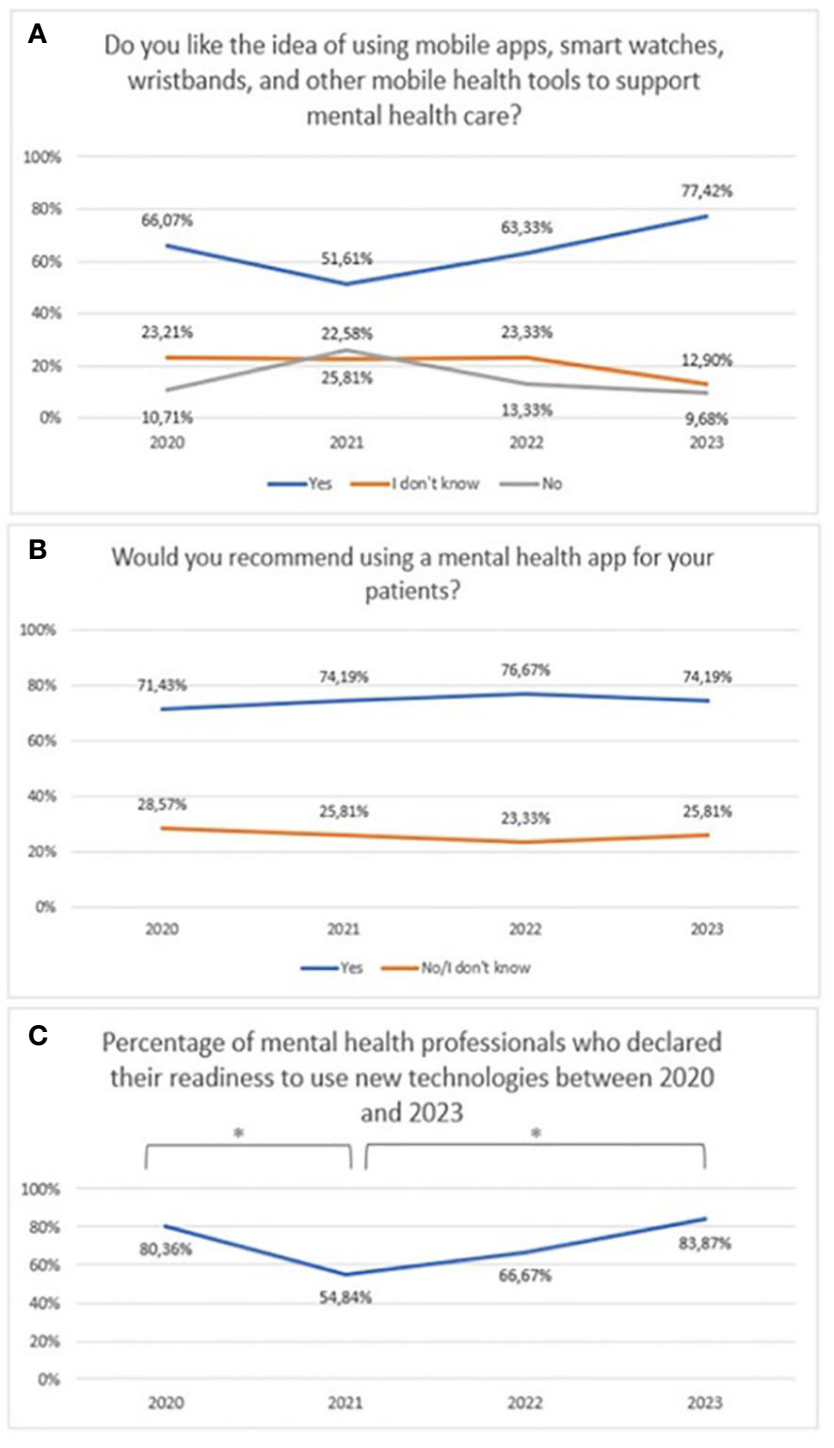
Responses regarding **(A)** idea of using mHealth tools over the period 2020-2023; **(B)** the declared desire recommend mental health applications over the period 2020-2023; **(C)** The percentage of mental health professionals who declared they were ready to use new technologies between 2020 and 2023. *statistically significant difference.

No statistically significant difference was claimed between 2020 and 2023 with regard to attitudes towards mHealth tools (χ^2^ = 26,72, df=6, p=0.348) (**Figure 7**).Over the period 2020-2023, a similar number of respondents declared a preference for recommending mental health apps to their patients (**Figure 7**). Responses of ‘No’ and ‘I don’t know’ were combined to ensure adequate group sizes were tested, but the chi-square test did not show statistical significance (χ^2^ = 0,29, df=3, p=0.961).The percentage of mental health professionals who declared they were ready to use new technologies between 2020 and 2023 is presented in **Figure 7** (χ^2^ = 9,19, df=3, p=0.027; statistically significance: 2020 vs 2021: χ^2^ = 6,35, df=1, p=0.012; 2021 vs 2023: χ^2^ = 6,15, df=1, p=0.013). A statistically significant increase was noted from 2021.

### 3.4 Concerns and risks towards telepsychiatry and mHealth tools in mental health care

#### 3.4.1 Concerns and risks associated with the use of telepsychiatry and mHealth tools in mental health care – aggregate analysis (2020–2023)

**Figure 8:**
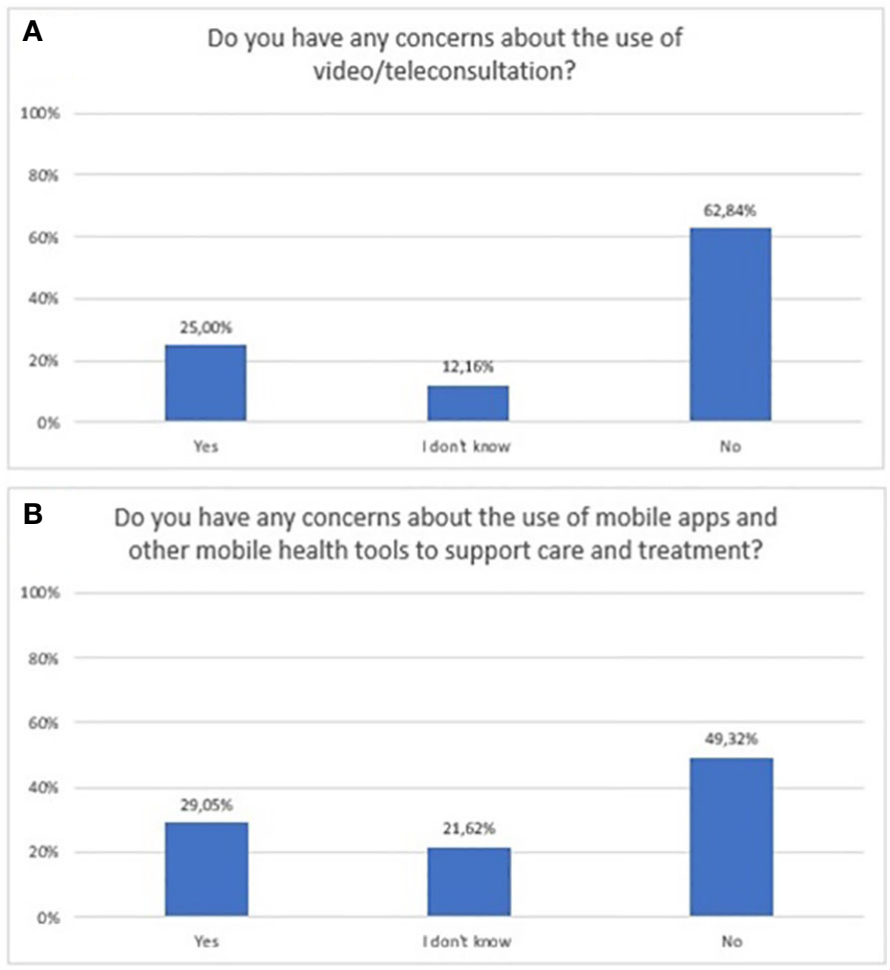
Responses regarding concerns and risks associated with the use of new technologies in mental health; **(A)** video/teleconsultation concerns; **(B)** mobile technologies concerns.

A quarter (n=37) and 29% (n=43) of respondents had some concern regarding the use of video/teleconsultation and mHealth tools, respectively. The exact distribution of responses to questions related to concerns over the use of new technologies is shown in **Figure 8**.

#### 3.4.2 Change in perceived concerns and risks associated with the use of telepsychiatry and mHealth tools in mental health care between 2020 and 2023

**Figure 9:**
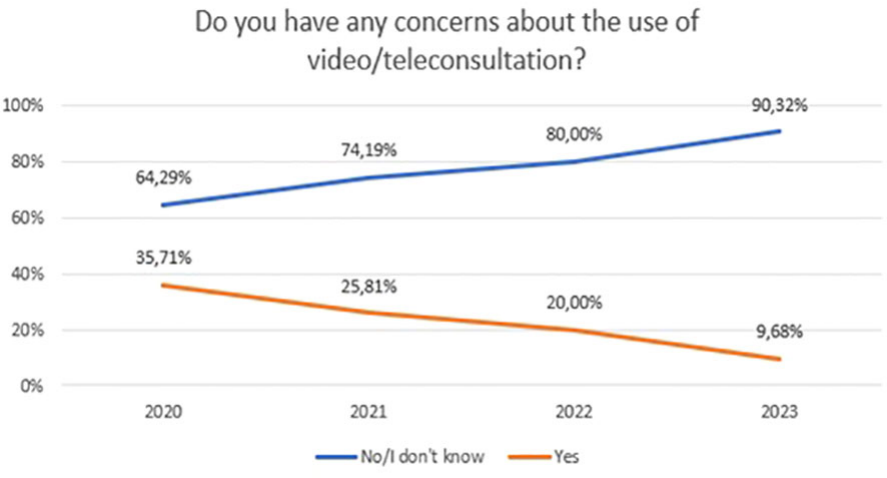
Responses regarding concerns about the use of video/teleconsultation over the period 2020-2023.

**Figure 10:**
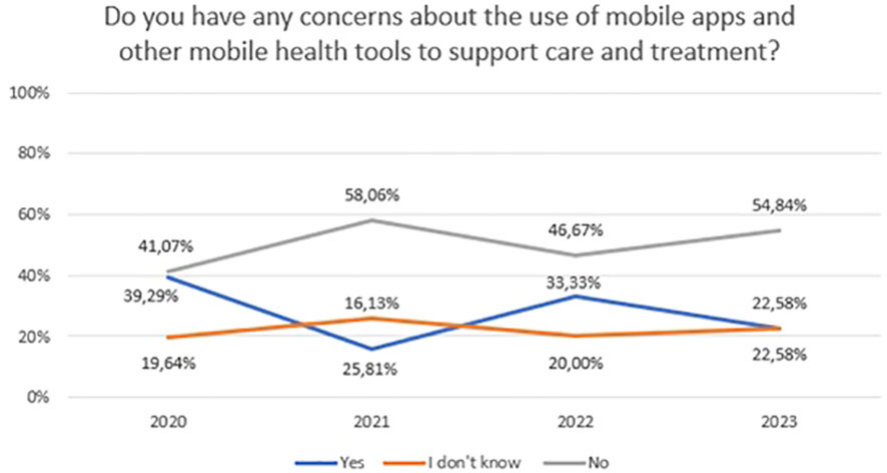
Responses regarding concerns about the use of mobile apps and other mobile health tools to support care and treatment over the period 2020-2023.

Concerns about video/teleconsultation and mobile technology in mental health between 2020 and 2023 are shown in **Figure 9** (χ^2^ = 7,72, df=3, p=0.052) and **Figure 10** (χ^2^ = 6,21, df=6, p=0.400). For **Figure 10**, the “No” and “I don’t know” responses have been summed to ensure sufficient group numbers to perform a chi-square test. The difference between years were not statistically significant.

### 3.5 Influence of age, profession, professional activity, workplace, and sex on readiness to use new technologies

The Kruskal-Wallis test showed that age (H=5,87; p=0.53), education (H=2,94; p=0.09), professional activity (H=1,87; p=0.17) and workplace (H=0,98; p=0.32) did not affect readiness to use new technologies. The Wilcoxon rank sum test showed that sex did not affect readiness to use new technologies (U=1936, p=0.58).

### 3.6 Analysis of open-ended responses

#### 3.6.1 Attitudes, expectations and preferences towards telepsychiatry and mHealth tools – analysis of open-ended questions (2020–2023)

In response to why respondents like the idea of video/teleconsultation as a tool to support the care of patients with mental disorders, the majority mentioned the possibility to contact a patient who cannot come for an appointment or is required by a pandemic situation (n=16). Three respondents described that it facilitates ongoing, continuous contact with the patient (n=3). In addition, 1 respondent each indicated that it improves the quality of patient care, support in disease monitoring, and “going with the times”. Among the respondents who did not like this idea, 4 indicated a preference for personal contact (n=4), one person indicated that such contact was difficult and exhausting, one described his doubts “it’s not for me”.

The majority of respondents who answered the question what they would like to improve indicated the quality of the call (n=10) and the need for a dedicated medical platform (n=6). In addition, they indicated confidentiality of the call and data protection (n=2), the possibility to assess wellbeing/risk of self-injurious actions, which could be visible to the therapist (n=1).

The participants gave a variety of responses as to why they liked the idea of using mobile technology, such as: to help monitor treatment and the therapeutic process (n=4), to help remission, support daily coping with the illness for chronically ill patients (n=3), to collect objective data (n=1), they can be an extra support for patients (n=1) and give the feeling of being taken care (n=1), to indicate that current apps are reliable and researched (n=2). Three respondents indicated that if they do not harm patients and they accept them then they should be used (n=3), while one respondent indicated that this “is the future”. Among those who did not like the idea, responses included inexperience and unfamiliarity with such apps (n=7), making long-term contact with the therapist more difficult (n=2), distracting patients from agency (n=1), driving them into an unreal world (n=1), and holding them as a ‘leash’ (n=1). Three respondents had mixed feelings about whether such solutions would help or harm patients (n=3).

#### 3.6.2 Concerns and risks associated with the use of telepsychiatry and mHealth tools – analysis of open-ended questions (2020–2023)

Among the concerns about teleconsultation in the open questions, respondents mentioned: difficulties in assessing the patient’s condition (n=7), inability to build an adequate therapeutic relationship (n=3), it is more tiring than face-to-face contact (n=3), contact with the patient is weaker (n=2), patients may give a feeling of being monitored (1), patients may want non-stop contact (n=1).

Among the concerns about mobile solutions in the open questions, respondents mentioned: lack of opportunity to try such effectiveness solutions, or not knowing which ones to recommend (n=8), security and privacy risk (n=6), mobile solutions may do more harm (n=2), mobile solutions may get boring (n=2), patient expectations may be too high (n=1), may delay contact with doctor (n=1). The detail results are given in **Supplementary Materials** (**Supplementary Material**).

## 4 Discussion

The growth of new technologies and digitalization is proceeding at a speed that is difficult for people, as well for science or legislation to keep up with. Moreover, it is not a temporary phenomenon, but rather an inevitable process, with any stabilization difficult to foresee. This expansion, especially in medical services, was greatly accelerated by the pandemic (43). Remote specialist-patient contact has become, in many areas of medicine, the only form of visits at the peak of the pandemic. This was undoubtedly a lever for telepsychiatry – currently a rapidly growing and constantly evolving branch of mHealth (6, 8). Clinical trials of new medical services and devices are struggling to follow the growth of the digital medical marketplace (44). These studies address both the effectiveness, safety and satisfaction of their users.

While much attention has been paid to the assessment and expectations of patients in this respect, the perspective of –clinicians remains under-researched (28). Studies to date have also had a short time frame, usually during a pandemic, which may not quite truly reflect attitudes in the absence of any other option for patient contact. Therefore, findings of this make a unique contribution to the literature on this rapidly evolving area, detailing clinicians’ attitudes and expectations towards digital technologies in mental health during and after Covid-19 outbreak.

Today, telepsychiatry delivered via tele/videoconferencing is an established and routinely used form of care delivery by mental health specialists (45). The efficacy of telepsychiatry is well documented in research (46). Its undoubted advantages include the ability to conduct a visit from anywhere, even a very remote location, easier appointment scheduling and easier rescheduling of appointments. Barriers identified for telepsychiatry include older age, lack of digital access, limited technical competence and hearing and visual impairment, and on the side of the professionals, the difficulty of convincing some of clinicians to use this form of consultation (13).

The APA and WPA have developed guidelines to provide a framework for ensuring the quality and safety of telepsychiatry (47, 48). It is worth highlighting at this point the difference that separates telepsychiatry and the rapidly growing digital health market, including online therapy provided by commercial firms. The latter, unlike telepsychiatry, has unproven security and is unregulated. This problem is evident in various countries, including Poland. An evaluation of this market in the United States revealed that most online therapy websites and applications are not regulated by the federal Health Insurance Portability and Accountability Act (HIPAA) (49). The paradox is that the same sensitive personal information protected by HIPAA when obtained by a doctor is not subject to any protection when obtained by mental health companies providing online therapy (50). These differences do not seem to be recognized by patients, so it is worth making them aware of this risk. This is especially true in terms of the security patients’ sensitive data, but also in terms of the unproven efficacy of the services offered by digital mental health companies.

Overall, patients’ and clinicians’ satisfaction with online consultations are rated highly. In recent survey, nine out of ten psychiatrists felt satisfied with telepsychiatry service (51). Previous satisfaction surveys on the use of tele/video consultation by mental health professionals, including those from the beginning of the pandemic, have also shown that this form of contact is well appreciated (32–34, 52–54). In this aspect, therefore, the results of our study are in line with those of the previous ones. In this survey, the willingness to use such a tool during work was expressed by up to 76% of respondents, with 70% of professionals stating that they use telepsychiatry at least once a week. In addition, however, our survey shows a longer time perspective and the changes that have occurred since the beginning of the pandemic, during its peak and with the end of the pandemic. A high level of interest was recorded especially in 2020-2021, while a decrease to 50% was recorded in 2021-2022 and then a slow rise again until 2023. One possible explanation could be a return to the traditional form of post-pandemic contact in those professionals who do however prefer the traditional form of contact with patient. It may be interesting to further observe this trend over time and investigate whether this is a temporary decline in interest, or whether some professionals will return to remote contact in the longer term. This will certainly be shaped from both sides i.e. patients and professionals. At the moment, however, there is a decrease in the declared intention to use video/tele-consultation from 2022, from more than 50% of all visits to less than half of all visits in 2023. It is also worth noting that a consistent proportion of professionals (25%) reported some concern about the use of video/teleconsultation over the 2020s to 2023. This may indirectly reflect a certain proportion of professionals for whom telepsychiatry will be a necessary choice rather than their preference.

Interestingly, in the open-ended questions, the study participants who had negative attitudes toward telepsychiatry most often indicated a preference for personal, traditional contact with the patient. Concerns about the use of telepsychiatry were mainly related to difficulties in correctly assessing the patient’s condition and establishing an appropriate therapeutic relationship. This is in line with the results of the survey from 2021-2022 (35). Professionals perceived telepsychiatry as a convenient solution for follow-up visits, however, it was perceived as less effective for setting up a therapeutic relationship or assessment of mental status in acute mental crisis. We also examined in our study what needs to be improved in telepsychiatry. Among the points indicated there were the quality of the connection and the lack of a platform dedicated to this solution.

In one study some gender differences have been detected, showing that women are more willing to use are more satisfied with the use of telepsychiatry (55). In this study, however, we found no differences regarding gender, age, or place of work.

A more constant and unchanged attitude over the years of the survey was presented by professionals regarding new mHealth tools, i.e. mobile apps, smart watches, monitoring wristbands, etc. More than half of the professionals were interested in the possibilities of mobile mental health aids, some have already recommended them to their patients. According to the majority of respondents (86%), mHealth tools can help patients to better cope with their mental illness and 74% declared recommending patients to use of mental health apps. This result is in general consistent with other identified studies (30, 34, 56, 57). Moreover, in a 2022 Portuguese survey as many as 87% clinicians supported the possibility of prescribing mental health apps (31). Although professionals’ attitudes about mHealth tools supporting patients may not necessarily translate into behavior, so much interest is encouraging. However, it is worth to mention that 29% of respondents had some concerns about the use of such devices by patients. These concerns were mainly related to the unknown effectiveness of these devices and doubts about the security of the data entered there by patients. In another study also pointed to technical problems encountered by clinicians and organizational and social factors related to concerns about implementing these solutions in daily practice (28). The problem of failing to protect sensitive user data that can threaten patient safety has been recognized for several years now. The business practices of digital companies are still not subject to proper scrutiny and may put profits ahead of security. Some authorities even suggest that specialists should screen apps before recommending them to patients (58).

Although regulations have already appeared in both the EU (59, 60) and the US (61) addressing this issue, they are still not perfect and most applications are not under the strict control of the relevant authorities. According to the 2017 EU Medical Device Regulation. Medical mobile apps require CE marking. In addition, the EU regulation has forced many app developers to improve privacy policy transparency. These regulations are expected to be clarified in the future, allowing the broad market for medical apps to be covered (62).

A good example of how to solve this problem is the German system. In 2019, a law was passed allowing doctors to prescribe certain health apps. In order to obtain such status, apps must undergo a comprehensive certification process and provide scientific evidence of efficacy and safety confirmed by clinical trials. Such certified apps are called DiGA, a specialist can prescribe it to a patient, and statutory health insurance covers the costs incurred. This seems to have solved both the problem of specialists unsure of what is effective and safe, as well as ensuring the security of sensitive patient data. This is confirmed by a willingness to use DiGA of as much as 76%. Therefore, it seems that it is not only the attitudes of clinicians to new technologies themselves that are important here, but also their confidence and knowledge based on evidence-based medicine, so that they can recommend such new solutions to their patients without any doubt. A multidisciplinary approach is needed to develop a tool that is effective and safe for patients. This requires the sound knowledge and experience of mental health professionals, a patient-assessed usability perspective, as well as the technical expertise of computer scientists. Although individual apps and smartwatches are being validated in clinical trials, the number of randomized clinical trials on large groups of patients is particularly low.

This study has also found that the use of internet-enabled mobile devices is widespread among mental health professionals, with as many as 92% using them at least once a week. Furthermore, over the period of the survey, there has been an increase in the percentage of professionals declaring that they feel ready to use new digital technologies in mental health, rising to 84% in 2023. This is generally in line with the data from the systematic review of this topic. These results allow us to look optimistically to the future and the development of medical devices and new channels for specialist-patient communication. However, the need to adapt legal solutions to the evolving digital market should not be overlooked, so that patient safety and the privacy of the patient’s sensitive data is not compromised.

This study has certain limitations that should be considered. Firstly, the mental health professionals who decided to participate in the survey may be predisposed to have positive attitudes toward telepsychiatry and m-health. Secondly, the response rate was 59.2%, which may have affected the results. Thirdly, most of the data came from mental health professionals working in urban areas and providing outpatient treatment. Lastly, the cross-sectional design and self-reported data were also limitations of this study. Therefore, the results should be generalized with particular care.

## 5 Conclusions

The study contributes to the body of knowledge on the attitudes, expectations and concerns of mental health professionals regarding the use of mobile digital technology and how these change over the onset, peak and extinction of the COVID-19 pandemic (2020–2023). The willingness to use telepsychiatry was expressed by up to 76% of respondents. However, since 2022 (pandemic extinction) there has been a decrease in the declared intention to use this modality from more than 50% to less than half of all visits in 2023. It is also worth noting that a consistent proportion of professionals (25%) reported some concern about the use of telepsychiatry, mainly related to difficulties in correctly assessing the patient’s condition. Furthermore, they indicated that technical issues such as the connection quality or special platform presence needed to be addressed.

According to the majority of respondents (86%), mHealth tools can help patients to better cope with their mental illness and 74% declared recommending patients their use. However, 29% of respondents had some concerns about the use of such devices by patients. These concerns were mainly related to the unknown effectiveness of these devices and doubts about the security of the data entered there by patients.

Finally, over the period of the survey, there has been an increase in the percentage of professionals declaring that they feel ready to use new digital technologies in mental health, rising to 84% in 2023. Determining concerns and expectations will enable the design of tools that are better suited and able to serve in the long term, rather than just being a short-term novelty.

## Data availability statement

The raw data supporting the conclusions of this article will be made available by the authors, without undue reservation.

## Ethics statement

The studies involving humans were approved by Bioethics Committee at the Institute of Psychiatry and Neurology, Warsaw, Poland. The studies were conducted in accordance with the local legislation and institutional requirements. Written informed consent for participation was not required from the participants or the participants’ legal guardians/next of kin in accordance with the national legislation and institutional requirements.

## Author contributions

MD: Conceptualization, Data curation, Formal analysis, Funding acquisition, Investigation, Methodology, Project administration, Writing – original draft, Writing – review & editing. AG: Formal analysis, Visualization, Writing – original draft, Writing – review & editing. AA: Funding acquisition, Supervision, Writing – review & editing. PM: Writing – review & editing, Conceptualization, Project administration, Supervision.

## References

[1] Hidalgo-Mazzei D Mateu A Reinares M Undurraga J Bonnin CD Sanchez-Moreno J. Self-monitoring and psychoeducation in bipolar patients with a smart-phone application (SIMPLe) project: design, development and studies protocols. BMC Psychiatry. (2015) 15:52. doi: 10.1186/s12888-015-0437-6 25884824 PMC4379950

[2] Vitger T Hjorthøj C Austin SF Petersen L Tønder ES Nordentoft M. A smartphone app to promote patient activation and support shared decision-making in people with a diagnosis of schizophrenia in outpatient treatment settings (Momentum trial): randomized controlled assessor-blinded trial. J Med Internet Res. (2022) 24:e40292. doi: 10.2196/40292 36287604 PMC9647453

[3] Torous J Larsen ME Depp C Cosco TD Barnett I Nock MK. Smartphones, sensors, and machine learning to advance real-time prediction and interventions for suicide prevention: a review of current progress and next steps. Curr Psychiatry Rep. (2018) 20:51. doi: 10.1007/s11920-018-0914-y 29956120

[4] Bufano P Laurino M Said S Tognetti A Menicucci D. Digital phenotyping for monitoring mental disorders: systematic review. J Med Internet Res. (2023) 25:e46778. doi: 10.2196/46778 38090800 PMC10753422

[5] Faurholt-Jepsen M Frost M Ritz C Christensen EM Jacoby AS Mikkelsen RL. Daily electronic self-monitoring in bipolar disorder using smartphones - the MONARCA I trial: a randomized, placebo-controlled, single-blind, parallel group trial. Psychol Med. (2015) 45:2691–704. doi: 10.1017/S0033291715000410 26220802

[6] Molfenter T Roget N Chaple M Behlman S Cody O Hartzler B. Use of telehealth in substance use disorder services during and after COVID-19: online survey study. JMIR Ment Heal. (2021) 8:e25835. doi: 10.2196/25835 PMC7895293 33481760

[7] Molfenter T Heitkamp T Murphy AA Tapscott S Behlman S Cody OJ. Use of telehealth in mental health (MH) services during and after COVID-19. Community Ment Health J. (2021) 57:1244–51. doi: 10.1007/s10597-021-00861-2 PMC8222700 34165695

[8] Goodyear T Richardson C Aziz B Slemon A Gadermann A Daly Z. Mental distress and virtual mental health resource use amid the COVID-19 pandemic: Findings from a cross-sectional study in Canada. Digit Heal. (2023) 9:20552076231173530. doi: 10.1177/20552076231173528 PMC10164262 37163172

[9] Hossain MM Tasnim S Sultana A Faizah F Mazumder H Zou L. Epidemiology of mental health problems in COVID-19: a review. F1000Research. (2020) 9:636. doi: 10.12688/f1000research.24457.1 33093946 PMC7549174

[10] Wu T Jia X Shi H Niu J Yin X Xie J. Prevalence of mental health problems during the COVID-19 pandemic: A systematic review and meta-analysis. J Affect Disord. (2021) 281:91–8. doi: 10.1016/j.jad.2020.11.117 PMC7710473 33310451

[11] Melcher J Hays R Torous J. Digital phenotyping for mental health of college students: a clinical review. Evid Based Ment Health. (2020) 23:161–6. doi: 10.1136/ebmental-2020-300180 PMC10231503 32998937

[12] Organization WH. mHealth: new horizons for health through mobile technologies. mHealth New horizons Heal through Mob Technol. (2011).

[13] Cowan KE McKean AJ Gentry MT Hilty DM. Barriers to use of telepsychiatry: clinicians as gatekeepers. Mayo Clin Proc. (2019) 94:2510–23. doi: 10.1016/j.mayocp.2019.04.018 31806104

[14] Matthews M Abdullah S Murnane E Voida S Choudhury T Gay G. Development and evaluation of a smartphone-based measure of social rhythms for bipolar disorder. Assessment. (2016) 23:472–83. doi: 10.1177/1073191116656794 PMC6155452 27358214

[15] Antosik-Wojcinska AZ Dominiak M Chojnacka M Kaczmarek-Majer K Opara KR Radziszewska W. Smartphone as a monitoring tool for bipolar disorder: a systematic review including data analysis, machine learning algorithms and predictive modelling. Int J Med Inform. (2020) 138:104131. doi: 10.1016/j.ijmedinf.2020.104131 32305023

[16] Weintraub MJ Posta F Ichinose MC Arevian AC Miklowitz DJ. Word usage in spontaneous speech as a predictor of depressive symptoms among youth at high risk for mood disorders. J Affect Disord. (2023) 323:675–8. doi: 10.1016/j.jad.2022.12.047 PMC9848879 36528134

[17] Dominiak M Kaczmarek-Majer K Antosik-Wójcińska AZ Opara KR Olwert A Radziszewska W. Behavioral and self-reported data collected from smartphones for the assessment of depressive and manic symptoms in patients with bipolar disorder: prospective observational study. J Med Internet Res. (2022) 24:e28647. doi: 10.2196/28647 34874015 PMC8811705

[18] Brian RM Ben-Zeev D. Mobile health (mHealth) for mental health in Asia: Objectives, strategies, and limitations. Asian J Psychiatr. (2014) 10:96–100. doi: 10.1016/j.ajp.2014.04.006 25042960

[19] Wilhelm K Handley T McHugh C Lowenstein D Arrold K. The quality of internet websites for people experiencing psychosis: pilot expert assessment. JMIR Form Res. (2022) 6:e28135. doi: 10.2196/28135 35436206 PMC9055477

[20] Lecomte T Potvin S Corbière M Guay S Samson C Cloutier B. Mobile apps for mental health issues: meta-review of meta-analyses. JMIR Mhealth Uhealth. (2020) 8:e17458. doi: 10.2196/17458 32348289 PMC7293054

[21] Carrouel F du Sartz de Vigneulles B Bourgeois D Kabuth B Baltenneck N Nusbaum F. Mental health mobile apps in the french app store: assessment study of functionality and quality. JMIR mHealth uHealth. (2022) 10:e41282. doi: 10.2196/41282 36223178 PMC9607929

[22] Daus H Kislicyn N Heuer S Backenstrass M. Disease management apps and technical assistance systems for bipolar disorder: Investigating the patients’ point of view. J Affect Disord. (2018) 229:351–7. doi: 10.1016/j.jad.2017.12.059 29331693

[23] Ben-Zeev D Brenner CJ Begale M Duffecy J Mohr DC Mueser KT. Feasibility, acceptability, and preliminary efficacy of a smartphone intervention for schizophrenia. Schizophr Bull. (2014) 40:1244–53. doi: 10.1093/schbul/sbu033 PMC4193714 24609454

[24] Faurholt-Jepsen M Busk J Frost M Vinberg M Christensen EM Winther O. Voice analysis as an objective state marker in bipolar disorder. Transl Psychiatry. (2016) 6:e856. doi: 10.1038/tp.2016.123 27434490 PMC5545710

[25] Antosik-Wójcinska A Chojnacka M Dominiak M Swiecicki Ł. The use of smartphones in the management of bipolar disorder-mobile apps and voice analysis in monitoring of mental state and phase change detection. Eur Neuropsychopharmacol. (2019) 29:S528–9. doi: 10.1016/j.euroneuro.2018.11.784

[26] Saunders KEA Bilderbeck AC Panchal P Atkinson LZ Geddes JR Goodwin GM. Experiences of remote mood and activity monitoring in bipolar disorder: A qualitative study. Eur Psychiatry. (2017) 41:115–21. doi: 10.1016/j.eurpsy.2016.11.005 PMC5947817 28135594

[27] Walsh S Golden E Priebe S. Systematic review of patients’ participation in and experiences of technology-based monitoring of mental health symptoms in the community. BMJ Open. (2016) 6:e008362. doi: 10.1136/bmjopen-2015-008362 PMC4916567 27329437

[28] Jacob C Sanchez-Vazquez A Ivory C. Social, organizational, and technological factors impacting clinicians’ Adoption of mobile health tools: systematic literature review. JMIR Mhealth Uhealth. (2020) 8:e15935. doi: 10.2196/15935 32130167 PMC7059085

[29] Dahlhausen F Zinner M Bieske L Ehlers JP Boehme P Fehring L. There’s an app for that, but nobody’s using it: Insights on improving patient access and adherence to digital therapeutics in Germany. Digit Heal. (2022) 8:20552076221104670. doi: 10.1177/20552076221104672 PMC9260569 35811758

[30] Mayer G Gronewold N Alvarez S Bruns B Hilbel T Schultz J-H. Acceptance and expectations of medical experts, students, and patients toward electronic mental health apps: cross-sectional quantitative and qualitative survey study. JMIR Ment Heal. (2019) 6:e14018. doi: 10.2196/14018 PMC6902133 31763990

[31] Nogueira-Leite D Diniz JM Cruz-Correia R. Mental health professionals’ Attitudes toward digital mental health apps and implications for adoption in Portugal: mixed methods study. JMIR Hum FACTORS. (2023) 10:e45949. doi: 10.2196/45949 37266977 PMC10276319

[32] Reliford A Zhang E Lanina O Williams SZ Sanichar N Khan S. Patient and clinician satisfaction with the early implementation of telemental health services in an urban behavioral health clinic during the COVID-19 pandemic. Telemed e-Health. (2023) 29(11) doi: 10.1089/tmj.2022.0480 36912813

[33] Gentry MT Puspitasari AJ McKean AJ Williams MD Breitinger S Geske JR. Clinician satisfaction with rapid adoption and implementation of telehealth services during the COVID-19 pandemic. Telemed E-HEALTH. (2021) 27:1385–92. doi: 10.1089/tmj.2020.0575 33606560

[34] Bucci S Berry N Morris R Berry K Haddock G Lewis S. “They are not hard-to-reach clients. We have just got hard-to-reach services.” Staff views of digital health tools in specialist mental health services. Front Psychiatry. (2019) 10:344. doi: 10.3389/fpsyt.2019.00344 31133906 PMC6524662

[35] Sheriff R Hong JSW Henshall C D’Agostino A Tomassi S Stein H. Evaluation of telepsychiatry during the COVID-19 pandemic across service users, carers and clinicians: an international mixed-methods study. BMJ Ment Heal. (2023) 26:e300646. doi: 10.1136/bmjment-2022-300646 PMC10577786 37567731

[36] Berry N Bucci S Lobban F. Use of the internet and mobile phones for self-management of severe mental health problems: qualitative study of staff views. JMIR Ment Heal. (2017) 4:e52. doi: 10.2196/mental.8311 PMC5688247 29092809

[37] Bondre AP Shrivastava R Raghuram H Tugnawat D Khan A Gupta S. A qualitative exploration of perceived needs and barriers of individuals with schizophrenia, caregivers and clinicians in using mental health applications in Madhya Pradesh, India. SSM Ment Heal. (2022) 2:100063. doi: 10.1016/j.ssmmh.2022.100063 PMC9792372 36688236

[38] Sinclair C Holloway K Riley G Auret K. Online mental health resources in rural Australia: clinician perceptions of acceptability. J Med Internet Res. (2013) 15:e193. doi: 10.2196/jmir.2772 24007949 PMC3785998

[39] Sarradon-Eck A Bouchez T Auroy L Schuers M Darmon D. Attitudes of general practitioners toward prescription of mobile health apps: qualitative study. JMIR mHealth uHealth. (2021) 9:e21795. doi: 10.2196/21795 33661123 PMC7974757

[40] Della Vecchia C Leroy T Bauquier C Pannard M Sarradon-Eck A Darmon D. Willingness of french general practitioners to prescribe mHealth apps and devices: quantitative study. JMIR mHealth uHealth. (2022) 10:e28372. doi: 10.2196/28372 35147508 PMC9491832

[41] Byambasuren O Beller E Glasziou P. Current knowledge and adoption of mobile health apps among Australian general practitioners: survey study. JMIR Mhealth Uhealth. (2019) 7:e13199. doi: 10.2196/13199 31199343 PMC6592476

[42] Association WM. World Medical Association Declaration of Helsinki: ethical principles for medical research involving human subjects. Jama. (2013) 310:2191–4. doi: 10.1001/jama.2013.28105355 24141714

[43] Golinelli D Boetto E Carullo G Nuzzolese AG Landini MP Fantini MP. Adoption of digital technologies in health care during the COVID-19 pandemic: systematic review of early scientific literature. J Med Internet Res. (2020) 22:e22280. doi: 10.2196/22280 33079693 PMC7652596

[44] Lau N O’Daffer A Colt S Yi-Frazier JP Palermo TM McCauley E. Android and iPhone mobile apps for psychosocial wellness and stress management: systematic search in app stores and literature review. JMIR mHealth uHealth. (2020) 8:e17798. doi: 10.2196/17798 32357125 PMC7275252

[45] APA. Telepsychiatry. (2022). Available at: https://www.psychiatry.org/psychiatrists/practice/telepsychiatry (Accessed September 10, 2023).

[46] O’Brien M McNicholas F. The use of telepsychiatry during COVID-19 and beyond. Ir J Psychol Med. (2020) 37:250–5. doi: 10.1017/ipm.2020.54 PMC7411439 32434596

[47] Mucic D Shore J Hilty DM. The world psychiatric association telepsychiatry global guidelines. J Technol Behav Sci. (2023) 64(S1):1–8. doi: 10.1007/s41347-023-00339-w

[48] Mishkind M Shore JH Barrett R Caudill R Chiu A Hilty D. Resource document on best practices in synchronous videoconferencing-based telemental health. Telemed e-Health. (2023). doi: 10.1089/tmj.2023.0174 38054938

[49] Feldstern N. Assessing anonymity: privacy in online mental healthcare and support groups. Tul J Tech Intell Prop. (2022) 24.

[50] Mueller R. Big data, big gap: working towards a HIPAA framework that covers big data. Indiana Law J. (2022) 97:1505.

[51] Torales J Vilallba-Arias J Bogado JA O’Higgins M Almirón-Santacruz J Ruiz Díaz N. Satisfaction with Telepsychiatry during the COVID-19 pandemic: Patients’ and psychiatrists’ report from a University Hospital. Int J Soc Psychiatry. (2023) 69:156–60. doi: 10.1177/00207640211070762 PMC9936176 34991382

[52] Kachnic J Wojtuszek M Wutke J Krysta K Krzystanek M. Telemedicine – How does it work in practice? Eur Psychiatry. (2017) 41:S147–8. doi: 10.1016/j.eurpsy.2017.01.1995

[53] Negev M Magal T Kaphzan H. Attitudes of psychiatrists toward telepsychiatry: A policy Delphi study. Digit Heal. (2023) 9. doi: 10.1177/20552076231177132 PMC10259121 37312951

[54] Roncero C Remon-Gallo D Casado-Espada N Aguilar L Gamonal-Limcaoco S Gallego MT. Healthcare professionals’ perception and satisfaction with mental health tele-medicine during the COVID-19 outbreak: A real-world experience in telepsychiatry. Front Psychiatry. (2022) 13:981346. doi: 10.3389/fpsyt.2022.981346 36405902 PMC9673754

[55] Luo J Tong L Crotty BH Somai M Taylor B Osinski K. Telemedicine adoption during the COVID-19 pandemic: gaps and inequalities. Appl Clin Inform. (2021) 12:836–44. doi: 10.1055/s-0041-1733848 PMC8426040 34496419

[56] Sterling WA Sobolev M Van Meter A Guinart D Birnbaum ML Rubio JM. Digital technology in psychiatry: survey study of clinicians. JMIR Form Res. (2022) 6:e33676. doi: 10.2196/33676 36355414 PMC9693695

[57] Bauer R Glenn T Monteith S Whybrow PC Bauer M. Survey of psychiatrist use of digital technology in clinical practice. Int J bipolar Disord. (2020) 8:29. doi: 10.1186/s40345-020-00194-1 33009954 PMC7532734

[58] Kazdin AE Rabbitt SM. Novel models for delivering mental health services and reducing the burdens of mental illness. Clin Psychol Sci. (2013) 1:170–91.

[59] R (EU). 2017/745 of the European parliament and of the council of 5 April 2017 on medical devices, amending Directive 2001/83/EC, Regulation (EC) No 178/2002 and Regulation (EC) No 1223/2009 and repealing Council Directives 90/385/EEC and 93/42/EEC (2017). Available at: https://eur-lex.europa.eu/legal-content/EN/TXT/?uri=CELEX%3A32017R0745.

[60] Regulation (EU) 2016/679 of the European Parliament and of the Council of 27 April 2016 on the protection of natural persons with regard to the processing of personal data and on the free movement of such data, and repealing Directive 95/46 . Available at: https://eur-lex.europa.eu/legal-content/EN/TXT/PDF/?uri=CELEX:32016R0679.

[61] Administration UF and D. US Department of Health and Human Services. Center for Devices and Radiological Health. Center for Biologics Evaluation and Research Food and Drug Administration. Policy for Device Software Functions and Mobile Medical Applications. Guidance. (2022). Available at: https://www.fda.gov/media/80958/download.

[62] Miralles I Granell C Díaz-Sanahuja L Van Woensel W Bretón-López J Mira A. Smartphone apps for the treatment of mental disorders: systematic review. JMIR mHealth uHealth. (2020) 8:e14897. doi: 10.2196/14897 32238332 PMC7163422

