# Supplementary material for: Mobile health for mental health support: a survey of attitudes and concerns among mental health professionals in Poland over the period 2020-2023
Source: Front Psychiatry. 2024 Mar 15;15:1303878. doi: 10.3389/fpsyt.2024.1303878 (PMC10978719; doi:10.3389/fpsyt.2024.1303878)
Supplement: Supplementary file 1 [file DataSheet_1.docx]

Supplementary Material

Mobile health for mental health support: a survey of attitudes and concerns among mental health professionals in Poland over the period 2020-2023

Monika Dominiak^1^, Adam Gędek^1,2*^, Anna Z. Antosik^3^, Paweł Mierzejewski^1^

^1^Department of Pharmacology, Institute of Psychiatry and Neurology, Warsaw, Poland

^2^Praski Hospital, Warsaw, Poland

^3^Department of Psychiatry, Faculty of Medicine, Collegium Medicum, Cardinal Wyszynski University in Warsaw, Poland

*** Correspondence:**Adam Gędek
adamgedek@gmail.com

# Supplementary Material – the full questionnaire

I. Prevalence and usage of new technologies

1. Do you use remote patients contact techniques, i.e. video or teleconsultation?
   1. Every day
   2. Often (at least once a week)
   3. Sometimes (at least once a month)
   4. Rare (once a year or less often)
   5. Never
2. Do you use internet-enabled mobile devices, i.e. a smartphone or tablet?
   1. Every day
   2. Often (at least once a week)
   3. Sometimes (at least once a month)
   4. Rare (once a year or less often)
   5. Never
3. Do you know that there are mobile apps, smart watches, wristbands, etc. which support mental health monitoring and help manage mental disorders daily?
   1. I know, I have recommended such solutions to patients
   2. I know, I am interested in the topic
   3. I know, but I am not interested
   4. I have heard a little
   5. I know nothing about it

II. Attitudes, expectations and preferences towards new technologies in psychiatry

1. Do you like the idea of using video/tele-consultation as a tool to support the care of patients with mental disorders?
   1. Yes
   2. I don’t know
   3. No
2. Why?
3. In what situations would you like to use video/tele-consultation?
   1. First visit
   2. As a complementary solution (alternating with traditional visits); As a continuation of treatment (subsequent visits)
   3. Only in exceptional situations where no other contact is possible
   4. Never
4. How often would you like to use video/tele-consultation in your work?
   1. More than 70% of visits
   2. 50-70% of visits
   3. 30-50% of visits
   4. less than 30% of visits
   5. never
5. What would you like to improve in the current video/tele-consultation tools, do you see a need for new functionalities, if so which ones?
6. Do you like the idea of using mobile apps, smart watches, wristbands and other mobile health tools to support mental health care?
   1. Yes
   2. I don’t know
   3. No
7. Why?
8. What features in a mental health mobile app do you think would be useful for patients?
   1. Educational - Psycho-educational materials to support management of the illness
      1. Yes
      2. No
   2. Self-motivating - Enabling the setting and achievement of goals
      1. Yes
      2. No
   3. Self-monitoring of well-being (e.g. sleep, mood, stress levels) with visualisation of this data e.g. in a chart and feedback
      1. Yes
      2. No
   4. Monitoring activity through e.g. call statistics, physical activity, mobility, voice parameters (to detect early signs of deterioration)
      1. Yes
      2. No
   5. Therapy support - Relaxation module
      1. Yes
      2. No
   6. Therapy support - Medication reminders
      1. Yes
      2. No
   7. Supporting therapy - Allowing on-going communication with the doctor/psychologist
      1. Yes
      2. No
9. Other features that you think would be helpful?
10. Would you recommend that your patients use a mental health app?
    1. Yes
    2. I don’t know
    3. No
11. In your opinion, could new technologies assist patients to better manage their mental disorders?
    1. Yes
    2. I don’t know
    3. No
12. Assess your readiness to use new technologies
    1. 1 (lack readiness)
    2. 2
    3. 3
    4. 4
    5. 5 (full readiness)
13. What other ways do you see new technologies being used in psychiatry ?

III. Concerns and risks associated with the use of new technologies in psychiatry

1. Do you have any concerns about the use of video/tele-consultation as a tool to support the care of patients with mental disorders?
   1. Yes
   2. I don’t know
   3. No
2. Which ones?
3. Do you have any concerns about the use of mobile apps, smart watches, wristbands and other mobile health tools to support mental health care?
   1. Yes
   2. I don’t know
   3. No
4. Which ones?
5. What other limitations/concerns do you see in the use of new technologies in psychiatry?

Metrics

1. Sex
   1. Female
   2. Male
2. Age
   1. 18-24 years
   2. 25-39 years
   3. 40-55 years
   4. 55-64 years
   5. >65years
3. Education
   1. Doctor
   2. Psychologist
   3. Psychotherapist
   4. Other:
4. Professional activity
   1. Hospital/psychiatric ward
   2. General hospital
   3. Outpatient clinic
   4. Other:
5. Workplace
   1. Village
   2. City up to 50,000
   3. City 50,000-250,000
   4. City >250,000
