# Supplementary material for: Mobile health for mental health support: a survey of attitudes and concerns among mental health professionals in Poland over the period 2020-2023
Source: Front Psychiatry. 2024 Mar 15;15:1303878. doi: 10.3389/fpsyt.2024.1303878 (PMC10978719; doi:10.3389/fpsyt.2024.1303878)
Supplement: Supplementary file 2 [file DataSheet_2.docx]

| **Question** | **Response / response categories** | **N (%)** |
| --- | --- | --- |
| Do you like the idea of using video/tele-consultation as a tool to support the care of patients with mental disorders? | Responses among participants who like the idea of using video/tele-consultation in mental health | 21 (77.8) |
|  | - the possibility to contact a patient who cannot come for an appointment or is required by a pandemic situation | 16 (59.3) |
|  | - facilitating continuous contact with the patient | 3 (11.1) |
|  | - improving the quality of patient care | 1 (3.7) |
|  | - support in disease monitoring, and “going with the times” | 1 (3.7) |
|  | Responses among participants who do not like the idea of using video/tele-consultation in mental health | 6 (22.2) |
|  | - preference for personal contact | 4 (14.8) |
|  | - such contact is difficult and exhausting | 1 (3.7) |
|  | - it is not for me | 1 (3.7) |
|  | All responses | 27 (100.0) |
| What would you like to see improved in the current video/tele-consultation tools,  do you see a need for new functionalities, if so which one? | - the quality of the call | 10 (52.6) |
|  | - the need for a dedicated medical platform | 6 (31.6) |
|  | - confidentiality of the call and data protection | 2 (10.5) |
|  | - the possibility to assess wellbeing/risk of self-injurious actions, which could be visible to the therapist | 1 (5.3) |
|  | All responses | 19 (100.0) |
| Do you like the idea of using mobile apps, smart watches, wristbands and other mobile health tools to support mental health care? Why? | Responses among participants who like the idea of using mobile health tools to support mental health care | 13 (46.5) |
|  | - they help monitor treatment and the therapeutic process | 4 (14.2) |
|  | - respondents indicated that if they do not harm patients and they accept them then they should be used | 3 (10.7) |
|  | - indicate that current apps are reliable and researched | 2 (7.1) |
|  | - enable to collect objective data | 1 (3.6) |
|  | - they can be an extra support for patients | 1 (3.6) |
|  | - can give a greater sense of being cared for | 1 (3.6) |
|  | - this “is the future” | 1 (3.6) |
|  | Responses among participants who do not like the idea of using mobile health tools to support mental health care | 12 (42.8) |
|  | - inexperience and unfamiliarity with such apps | 7 (25.0) |
|  | - might make long-term contact with the therapist more difficult | 2 (7.1) |
|  | - it draws attention away from own causality to something else | 1 (3.6) |
|  | - driving patient into an unreal world | 1 (3.6) |
|  | - sounds like a kind of leash | 1 (3.6) |
|  | Participants who have mixed feelings about whether such solutions would help or harm patients | 3 (10.7) |
|  | All responses | 28 (100.0) |
| Do you have any concerns about the use of video/tele-consultation as a tool to support the care of patients with mental disorders? | - difficulties in assessing the patient's condition | 7 (41.2) |
|  | - inability to build an adequate therapeutic relationship | 3 (17.6) |
|  | - it is more tiring than face-to-face contact | 3 (17.6) |
|  | - contact with the patient is weaker | 2 (11.8) |
|  | - patients may give a feeling of being monitored | 1 (5.9) |
|  | - patients may want non-stop contact | 1 (5.9) |
|  | All responses | 17 (100.0) |
| Do you have any concerns about the use of mobile apps, smart watches, wristbands and other mobile health tools to support mental health care? | - lack of opportunity to try such effectiveness solutions, or not knowing which ones to recommend | 8 (40.0) |
|  | - security and privacy risk | 6 (30.0) |
|  | - mobile solutions may do more harm | 2 (10.0) |
|  | - mobile solutions may get boring | 2 (10.0) |
|  | - patient expectations may be too high | 1 (5.0) |
|  | - may delay contact with a specialist | 1 (5.0) |
|  | All responses | 20 (100.0) |
